# CeSTAAN: An atlas of *C. elegans* adult neurons for fast queries of single-nucleus RNA sequencing data

**DOI:** 10.17912/micropub.biology.001785

**Published:** 2025-11-25

**Authors:** Jonathan St. Ange, Henry Langmack, Coleen T. Murphy

**Affiliations:** 1 LSI Genomics, Molecular Biology, Princeton University, Princeton, New Jersey, United States; 2 Princeton High School, Princeton New Jersey

## Abstract

RNA-sequencing provides rich transcriptomic data about cell types, and new methods allow characterization at the single-cell level. For neurons, single-nucleus RNA sequencing (snSeq) is the gold standard, as this technique retains neuron identity. We carried out snSeq on sorted nuclei from *C. elegans* neurons of Day 1 adult wild-type ( N2 ) and *daf-2* mutants, and males and genotypically-matched hermaphrodites. To provide the field with an easily accessible atlas of this information, we created *C. elegans* Single-nucleus Transcriptomic Atlas of Adult Neurons (CeSTAAN) (https://cestaan.princeton.edu/), described here with examples. CeSTAAN will allow the *C. elegans* field to access this adult neuron transcriptional information.

**Figure 1. CeSTAAN Website functionalities:**
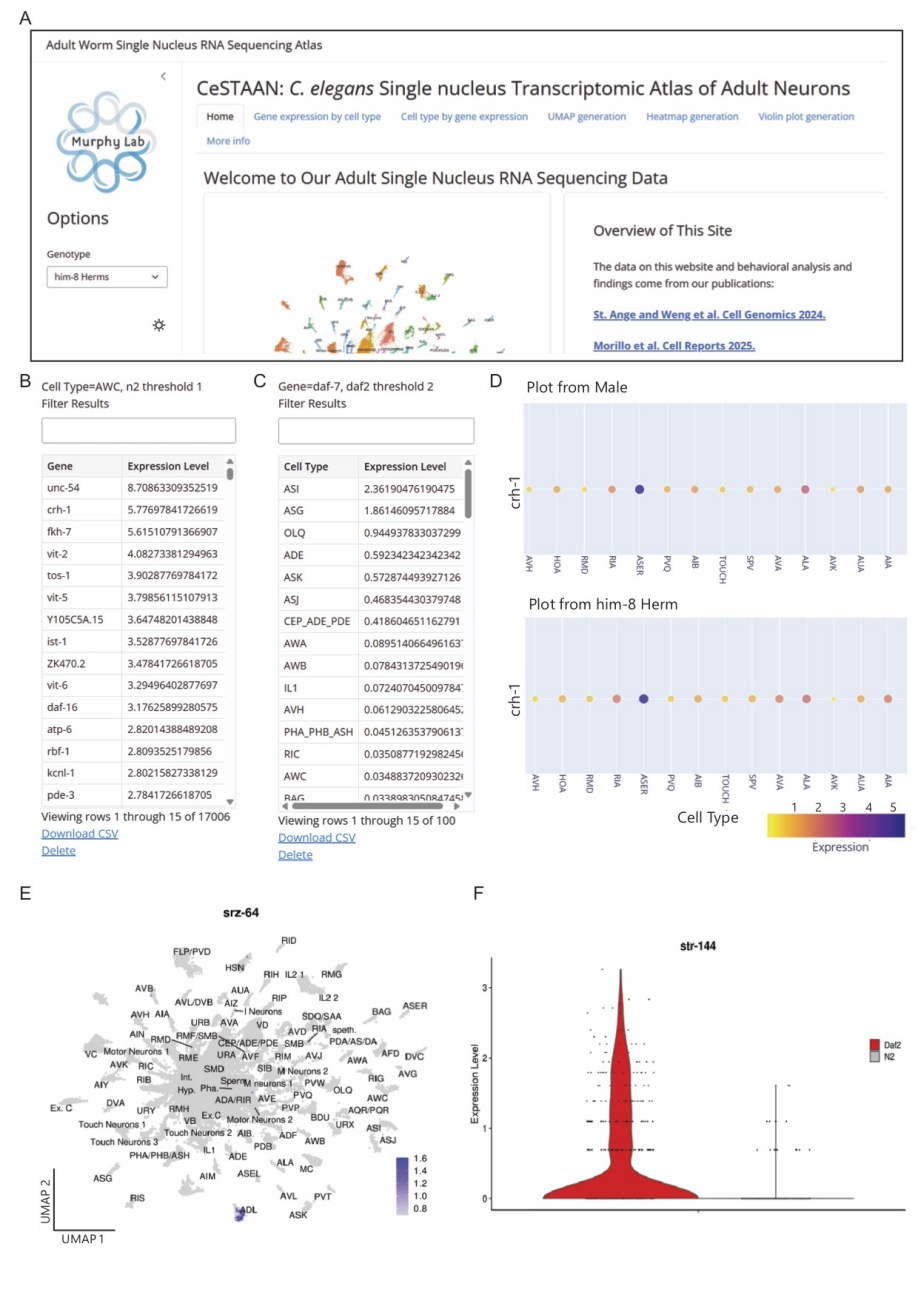
**A)** An excerpt from the homepage of the CeSTAAN website. On the side panel, the user can select the genotype or sex they would like to see transcriptomic information for as well as click between functionality tabs. These tabs allow for various user queries. **B)** ‘Gene Expression by Cell Type' tab allows the user to input a cell type and receive a downloadable gene expression table. **C)** ‘Cell Type by Gene Expression' tab allows the user to input a gene and receive a downloadable table of that gene's expression level across the nervous system. The website is also capable of plot generation, e.g., **D)** Excerpts from the interactive heatmap tab containing information on expression levels and percent expression of genes in all neurons captured in these data sets. The user can also input a gene or multiple genes to generate **E)** UMAP plots or **F)** violin plots from the data. Dot size is correlated with fraction of expressing cells in a given cluster.

## Description

The data available on CeSTAAN comes from our recent manuscripts using the single-nucleus RNA sequencing technique, as shown in St. Ange & Weng et al. (2024) ^1^ and Morillo et al. (2025) ^2^ .

Shiny python serves as the main structure for the website, while SQL databases quickly serve queries back to the user and R generates visuals for presentations if the user desires. The user can pick from several different tabs: Gene expression by cell type, Cell type by gene expression, UMAP generation, Heatmap generation, Violin plot generation, and more info. The output of the expression tabs are dynamic searchable tables that are available for any genotype or sex at any of four different gene expression and percent cell expression thresholds. UMAPs and Violin Plots generate from the .rds file containing the Seurat objects ^3^ and running R script. Finally, the Heatmap generation tab uses the SQL databases and python to generate an interactive heatmap that the user can zoom in and out of.

All plots and tables generated by the website are downloadable, and the more info tab contains a download button for our Seurat object files that are the fully processed objects from our papers.

## Methods

All datasets used for this site as well as scripts used to create the site are available at: https://github.com/svenmh/murphy-lab-project . These datasets come from St. Ange & Weng et al. (2024) as well as Morillo et al. (2025). All generation, processing, and quality control metrics for these datasets are available in their respective manuscripts. The raw read sequence data from these manuscripts are available on NCBI as BioProjects PRJNA1027859 and PRJNA1195922 respectively.

The expression values reported on the website are normalized Single-Cell Transform (SCT) expression values. SCT is a standard normalization process for single-cell data that addresses the large amount of zeros in the data and the variation between cells ^4^ . SCT values are very small where an average expression value in the N2 / *daf-2* dataset is ~0.05 and an average expression value in the male/hermaphrodite dataset is ~0.03. Importantly, however, these datasets were not all normalized, together so adult N2 animals can only be compared to the genotypically- and age-matched *daf-2* animals, while adult male data can only be compared to the genotypically- and aged-matched hermaphrodites.

The data for the website are stored as SQL databases containing average normalized expression values and percent expression values for each dataset. These databases are generated from excel sheets using a python script, which are all uploaded on github. For plot generation, the main Seurat objects are used. RDS files containing Seurat objects for the Adult Day 1 N2 / *daf-2* dataset and the Adult genotypically-matched male/hermaphrodite datasets are also on github. These files are opened using R, and PNGs are generated and handed back to the user. The main website uses a shiny python script as the interface for user input and reactive tables and plots. It connects to python and R files to respond to user inputs and quickly serve query results back to the user.

## Reagents

**Table:** 

Software/Tool	Website	Version	Publication reference
Python	https://www.python.org/	v3.9.21	-
R	https://www.r-project.org/	v4.5.0	-
SQLite	https://www.sqlite.org/	v3.34.1	-
Seurat	https://satijalab.org/seurat/	V4.4.0	(Hao et al. 2021)
Shiny for Python	https://shiny.posit.co/py/	v1.4.0	-

## References

[R1] Hafemeister C, Satija R (2019). Normalization and variance stabilization of single-cell RNA-seq data using regularized negative binomial regression.. Genome Biol.

[R2] Hao Y, Hao S, Andersen-Nissen E, Mauck WM 3rd, Zheng S, Butler A, Lee MJ, Wilk AJ, Darby C, Zager M, Hoffman P, Stoeckius M, Papalexi E, Mimitou EP, Jain J, Srivastava A, Stuart T, Fleming LM, Yeung B, Rogers AJ, McElrath JM, Blish CA, Gottardo R, Smibert P, Satija R (2021). Integrated analysis of multimodal single-cell data.. Cell.

[R3] Morillo KS, St Ange J, Weng Y, Kaletsky R, Murphy CT (2025). Single-nucleus neuronal transcriptional profiling of male C. elegans uncovers regulators of sex-specific and sex-shared behaviors.. Cell Rep.

[R4] St Ange J, Weng Y, Kaletsky R, Stevenson ME, Moore RS, Zhou S, Murphy CT (2024). Adult single-nucleus neuronal transcriptomes of insulin signaling mutants reveal regulators of behavior and learning.. Cell Genom.

